# Androgen Receptor Expression and Association With Distant Disease-Free Survival in Triple Negative Breast Cancer: Analysis of 263 Patients Treated With Standard Therapy for Stage I-III Disease

**DOI:** 10.3389/fonc.2019.00452

**Published:** 2019-06-06

**Authors:** Maria Vittoria Dieci, Vassilena Tsvetkova, Gaia Griguolo, Federica Miglietta, Mara Mantiero, Giulia Tasca, Enrico Cumerlato, Carlo Alberto Giorgi, Tommaso Giarratano, Giovanni Faggioni, Cristina Falci, Grazia Vernaci, Alice Menichetti, Eleonora Mioranza, Elisabetta Di Liso, Simona Frezzini, Tania Saibene, Enrico Orvieto, Valentina Guarneri

**Affiliations:** ^1^Department of Surgery, Oncology and Gastroenterology, University of Padova, Padova, Italy; ^2^Medical Oncology 2, Istituto Oncologico Veneto IOV-IRCCS, Padova, Italy; ^3^Anatomy and Histology Unit, Padova Hospital, Padova, Italy; ^4^Breast Surgery, Istituto Oncologico Veneto IOV-IRCCS, Padova, Italy; ^5^Pathology, Ulss 5 Polesana, Rovigo, Italy

**Keywords:** androgen receptor, triple negative, early breast cancer, androgen receptor, triple negative, early breast cancer, prognosis, late outcome

## Abstract

**Background:** We evaluated immunohistochemical AR expression and correlation with prognosis in a large series of homogeneously treated patients with primary TNBC.

**Material and Methods:** Patients diagnosed with stage I-III TNBC between 2000 and 2015 at Istituto Oncologico Veneto who received treatment with surgery and neoadjuvant and/or adjuvant chemotherapy were included. Whole tissue slides were stained for AR. AR-positive expression was defined as >1% of positively stained tumor cells. Distant-disease-free survival (DDFS) was calculated from diagnosis to distant relapse or death. Late-DDFS was calculated from the landmark of 3 years after diagnosis until distant relapse or death.

**Results:** We included 263 primary TNBC patients. Mean AR expression was 14% (range 0–100%), and 29.7% (*n* = 78) of patients were AR+. AR+ vs. AR- cases presented more frequently older age (*p* < 0.001), non-ductal histology (*p* < 0.001), G1-G2 (*p* = 0.003), lower Ki67 (*p* < 0.001) and lower TILs (*p* = 0.008). At a median follow up of 81 months, 23.6% of patients experienced a DDFS event: 33.3% of AR+ and 19.5% of AR- patients (*p* = 0.015). 5 years DDFS rates were 67.2% and 80.6% for AR+ and AR- patients (HR = 1.82 95%CI 1.10–3.02, *p* = 0.020). AR maintained an independent prognostic role beyond stage, but when TILs were added to the model only stage and TILs were independent prognostic factors. AR was the only factor significantly associated with late-DDFS: 16.4% of AR+ and 3.4% of AR- patients experienced a DDFS after the landmark of 3 years after diagnosis (*p* = 0.001). Late-DDFS rates at 5 years from the 3-year landmark were 75.8% for AR+ and 95.2% for AR- patients (log-rank *p* < 0.001; HR = 5.67, 95%CI 1.90–16.94, *p* = 0.002).

**Conclusions:** AR expression is associated with worse outcome for patients with TNBC. In particular, AR+ TNBC patients are at increased risk of late DDFS events. These results reinforce the rationale of AR targeting in AR+ TNBC.

## Introduction

Triple negative breast cancer (TNBC) represents the most lethal breast cancer subtype, accounting for around 15% of all breast cancer diagnoses and being associated with an increased risk of relapse at distant sites, mostly occurring within the first 3 years from diagnosis (1). It is defined by the absence of expression of estrogen and progesterone receptors and lack of HER-2 overexpression/amplification. To date, chemotherapy remains the mainstay of systemic treatment for TNBC, since no relevant druggable targets have been identified (2).

In recent years, the application of genomic profiling techniques has allowed to dissect the heterogeneity of TNBC. At least four main TNBC subtypes have been defined (3, 4), including the luminal androgen receptor (LAR) class, which is enriched for hormonally regulated pathways and is dependent on AR signaling. The LAR subtype accounts for approximately 10–15% of TNBC and LAR cell lines have shown sensitivity to AR-antagonists (3, 4).

AR is found to be expressed by immunohistochemistry in 60–80% of breast cancers, less frequently in estrogen receptor-negative as compared to estrogen-receptor positive tumors (5). In TNBC series, the rate of AR-positive cases is generally 20–40% (5–8), with few studies showing rates up to 60% (9). Preclinical evidence shows that the AR effect depends on tumor subtype: in estrogen receptor-positive cancer cells AR activity is able to inhibit tumor growth (10), whereas in TNBC AR seems to retain an oncogenic effect (11, 12). With regards to the prognostic role of AR expression in patients cohorts, available evidence supports an association between AR expression and favorable prognosis for estrogen receptor-positive tumors (5, 13). In TNBC, data are more conflicting, with some studies showing a favorable prognosis associated with AR expression, some showing null results and others showing an association between AR expression and unfavorable outcome (5). Different methods of AR assessment and scoring, heterogeneity in patients cohorts and short follow up may have yielded to these contrasting results.

In this study, we evaluated AR expression by immunohistochemistry and its correlation with distant disease-free survival in a large cohort of patients with non-metastatic TNBC homogeneously treated with surgery and systemic chemotherapy.

## Methods

### Patients Population

We included 263 patients with non-metastatic TNBC (estrogen receptor and progesterone receptor <10%, HER2 0/1+ by immunohistochemistry and/or FISH non amplified) diagnosed from March 2000 to December 2015 at IRCCS Istituto Oncologico Veneto (Padova, Italy) who received treatment with surgery and neoadjuvant and/or adjuvant chemotherapy. Clinicopathological characteristics as well as treatment and follow up data were collected in a dedicated database. The study protocol was approved by the Ethical Committee of the Istituto Ocologico Veneto IRCCS (Padova, Italy). Written informed consent was obtained from patients.

### Pathology Assessments

AR expression was evaluated on the following FFPE primary tumor samples for main analyses: surgical sample for patients treated with primary surgery followed by adjuvant chemotherapy and diagnostic core-biopsy for patients treated with neoadjuvant chemotherapy followed by surgery.

In case of patients treated with neoadjuvant chemotherapy showing residual invasive breast cancer at the examination of the surgical sample, the FFPE surgical tumor block was also retrieved in order to conduct exploratory analysis of changes in AR expression from pre- to post-neoadjuvant chemotherapy.

AR nuclear staining was evaluated on whole sections by immunohistochemistry with the Dako AR441 antibody. AR was scored by a dedicated pathologist, blinded for clinical data, and was considered positive in case of staining in at least 1% of tumor cells, consistently with most recent studies (8, 9).

Tumor infiltrating lymphocytes (TILs) were evaluated according to consensus guidelines on hematoxylin and eosin-stained slides (14).

### Statistical Analysis

Statistical analysis was carried out using IBM SPSS (version 24) software.

Descriptive statistics were performed for patient demographics and clinical characteristics. For continuous variables, median and quartiles were computed. The χ^2^ test or the Mann-Whitney non-parametric test were used to study association between variables, according to their nature (categorical or continuous). The Wilcoxon signed-rank test was used to study the changes in AR expression before and after neoadjuvant chemotherapy in the subset of patients who received this type of treatment and showing residual invasive disease on the surgical sample.

Distant-disease free survival (DDFS) was defined as the time from diagnosis to relapse at a distant site or death from any cause, whichever first. Late-DDFS analysis were performed from the landmark of 3 years after diagnosis until relapse at a distant site or death from any cause, whichever first. In late-DDFS analysis, patients with an event or censored before the landmark point were excluded. The landmark for late-DDFS was defined based on the pattern of relapse for TNBC that shows a peak in the hazard rate of recurrence in the first 3 years after diagnosis (15). Overall survival (OS) was defined as the time from diagnosis to death from any cause. The Kaplan–Meier method was used to estimate survival curves, the log-rank test was used to test difference between groups. Univariate and multivariate Cox regression models were used to calculate HR and 95% CI. All reported *p*-values are two-sided, and significance level was set at *p* < 0.05.

## Results

### Clinicopathological Characteristics and Association With AR

**Figure 1:**
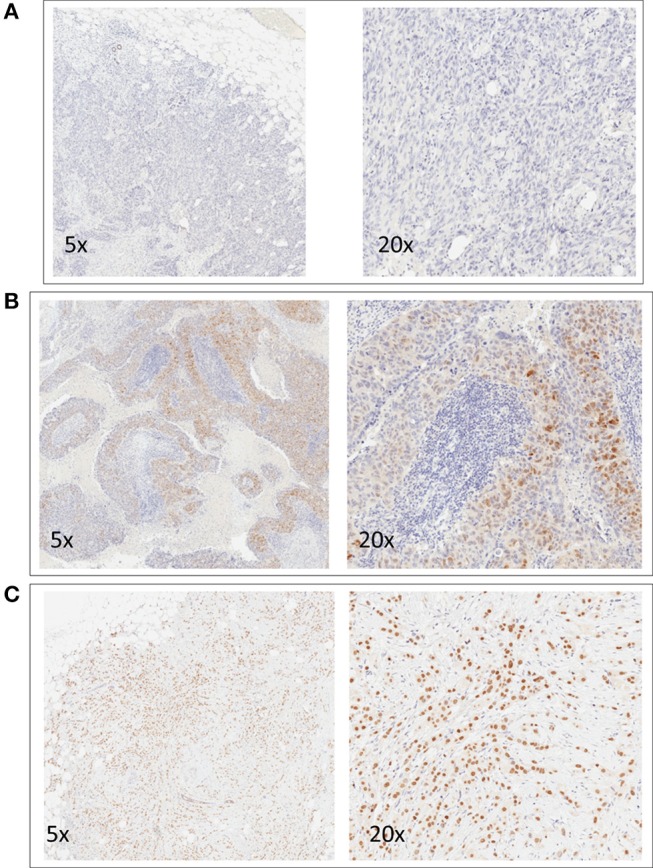
Representative images of immunohistochemical nuclear staining for AR. For each case, two images at different magnification are shown (5x and 20x). One negative case **(A)** and two positive cases **(B,C)** are shown.

Mean AR expression level was 14% (range 0–100%). Of 263 TNBC patients, 29.7% (*N* = 78) showed a positive AR expression. Images of representative slides are shown in Figure 1. Clinicopathological characteristics according to AR status are reported in Table 1. AR expression was significantly associated with older age (*p* = 0.002), non-ductal histology (*p* < 0.001), Grade 1–2 tumors (*p* = 0.003), lower Ki67 (*p* < 0.001), lower TILs (*p* = 0.008). There was no difference in stage and treatment received according to AR. Considering neoadjuvant and adjuvant therapy combined, 73% of patients received both an anthracycline and a taxane as part of chemotherapy treatment.

**Table 1:** Clinicopathological patients' characteristics by AR expression.

**Patients' features**		**ALL (*N* = 263) *N* (%)**	**AR+ (*N* = 78) *N* (%)**	**AR- (*N* = 185) *N* (%)**	***p***
Age years, median (Q1-Q3)		53 (44–66)	62 (47–70)	51 (42–62)	0.002
Hystotype	Ductal/NOS	237 (91.2)	66 (84.6)	171 (94.0)	
	Lobular	7 (2.7)	5 (6.4)	2 (1.1)	
	Apocrine	8 (3.1)	7 (9.0)	1 (0.5)	
	Metaplastic	5 (1.9)	0	5 (2.7)	
	Medullary	3 (1.2)	0	3 (1.6)	<0.001
AJCC Stage	I	83 (31.7)	24 (30.8)	59 (32.1)	
	II	130 (49.6)	38 (48.7)	92 (50.0)	
	III	49 (18.7)	16 (20.5)	33 (17.9)	0.886
Grade	G1-2	29 (11.9)	16 (21.1)	13 (7.7)	
	G3	2515 (88.1)	60 (78.9)	155 (92.3)	0.003
Ki67%, median (Q1-Q3)		55 (36–70)	40 (27–60)	60 (40–70)	<0.001
TILs%, median (Q1-Q3)		10 (5–30)	7 (2–20)	10 (5–30)	0.008
Neoadjuvant CT	Yes	108 (41.1)	27 (34.6)	81 (43.8)	
	No	155 (58.9)	51 (65.4)	104 (56.2)	0.167
Type of neoadjuvant CT	Anthra+tax	101 (93.5)	24 (88.9)	77 (95.1)	
	Anthra	1 (0.9)	0	1 (1.2)	
	Tax	6 (5.6)	3 (11.1)	3 (3.7)	0.299
Adjuvant CT	Yes	186 (71.3)	57 (73.1)	129 (70.5)	
	No	75 (28.7)	21 (26.9)	54 (29.5)	0.673
Type of adjuvant CT	Anthra+tax	96 (51.6)	28 (49.1)	68 (52.7)	
	Anthra	37 (19.9)	14 (24.6)	23 (17.8)	
	Tax	8 (4.3)	1 (1.8)	7 (5.4)	
	Other	45 (24.2)	14 (24.6)	31 (24.0)	0.523
Radiotherapy	Yes	165 (67.1)	48 (64.9)	117 (68.0)	
	No	81 (32.9)	26 (35.1)	55 (32.0)	0.629

*N, number, AR, androgen receptor; p, p-value; Q1, first quartile; Q3, third quartile; NOS, not otherwise specified; AJCC, American Joint Committee on Cancer; TILs, tumor infiltrating lymphocytes; CT, chemotherapy; Anthra, anthracycline; Tax, taxane*.

### Survival Analyses

At a median follow up of 81 months (95% CI 74–87), 62 patients have experienced a DDFS event (23.6%). Type of DDFS event was: distant relapse in 56 patients (90%) and death in 6 patients (10%, two deaths occurred in patients with unresectable chest locoregional recurrence and 4 patients died without known breast cancer relapse). The rate of events was higher in AR+ as compared to AR- patients (33.3 and 19.5%, respectively).

**Figure 2:**
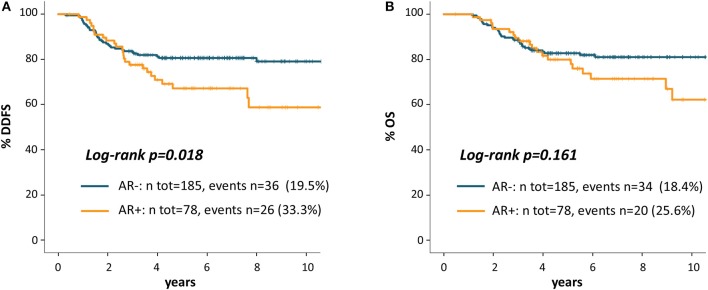
Kaplan Meier curves for distant disease-free survival **(A)** and overall survival **(B)** according to AR.

As shown in Figure 2A, Patients with AR+ tumor showed worse DDFS as compared to AR- patients: 5 years DDFS rates were 67.2 and 80.6%, respectively (log-rank *p* = 0.018). The HR for DDFS for the comparison of AR+ vs. AR- groups was 1.82 (95%CI 1.10-3.02, *p* = 0.020).

Figure 2B shows OS Kaplan-Meier curves: 5 years OS rate was 79.9% for AR+ and 82.7% for AR- patients (log-rank *p* = 0.161). The HR for OS for the comparison of AR+ vs. AR- patients was 1.48 (95% CI 0.85-2.58, *p* = 0.163).

Univariate and multivariate cox models for DDFS are reported in Table 2.

**Table 2:** Univariate and multivariate DDFS cox models.

	**Univariate**			Multivariate model 1^*^			Multivariate model 2^**^		
	**HR**	**95%CI**	***p***	**HR**	**95%CI**	***p***	**HR**	**95%CI**	***p***
Age (continuous)	1.01	0.90–1.03	0.446	–	–	–	–	–	–
Grade 1-2	Ref			–	–	–	–	–	–
Grade 3	1.29	0.55–3.02	0.553						
Stage I	Ref			Ref			Ref		
Stage II-III	2.07	1.10–3.89	0.024	3.05	1.83–5.08	<0.001	2.34	1.26–4.47	0.008
TILs (1% increments)	0.98	0.96–0.99	0.005	–	–	–	0.98	0.96–0.99	0.004
AR –	Ref			Ref			Ref		
AR +	1.82	1.10–3.02	0.020	1.74	1.05–2.88	0.032	1.57	0.94–2.61	0.084

*HR, hazard ratio; CI, confidence interval; p, p-value; TILs, tumor infiltrating lymphocytes, AR, androgen receptor*.

* *Including stage and AR*.

** *Including stage, AR and TILs*.

In addition to AR, the other factors that were associated in univariate analysis with DDFS, were stage (Stage II-III vs. I, *p* = 0.024) and TILs (considered as continuous variable for each 1% increment, *p* = 0.005). In multivariate analysis including AR and Stage, both factors maintained an independent prognostic role (AR+ vs. AR-: HR = 1.74, 95%CI 1.05-2.88, *p* = 0.032; Stage II-III vs. I: HR 3.05, 95%CI 1.83-5.08, *p* < 0.001). When TILs were added to the multivariate model, only stage and TILs maintained an independent prognostic value. The HR for the association between AR status and DDFS in multivariate models including the three variables was 1.57 (95% CI 0.94-2.61, *p* = 0.084).

Since Kaplan Meier curves showed that the prognostic effect of AR on DDFS appeared driven by the occurrence of late recurrences in AR+ patients, we performed a landmark survival analysis for late-DDFS to study the association between AR and late outcome. This analysis included 203 patients who were DDFS-free at 3 years from initial diagnosis and were not censored before the landmark point: *n* = 55 (27%) were AR+ and *n* = 148 (73%) were AR-. At a median follow up of 47 months (95% CI 41-53) *n* = 14 DDFS events have occurred. The rate of event was higher in AR+ (9/55, 16.4%) vs. AR- patients (5/148, 3.4%). Type of DDFS event included: 10 distant relapses and 4 deaths (1 in a patient with unresectable locoregional breast recurrence and 3 in patients without prior known breast cancer relapse). AR+ patients showed more frequently distant relapses (*n* = 8 of 9 total events, 89%) as compared to AR- patients (*n* = 2 of 5 total events, 40%).

**Figure 3:**
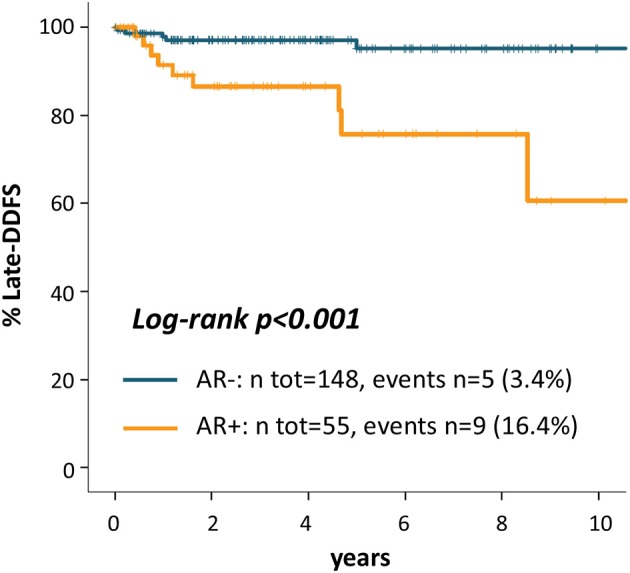
Kaplan Meier curves for late-distant disease-free survival from the landmark of 3 years after diagnosis according to AR.

Kaplan Meier curves in Figure 3 shows that patients with AR+ tumor experienced a significantly worse late outcome as compared to AR- patients: late-DDFS rate at 5 years from the 3-years landmark were 75.8% for AR+ patients and 95.2% for AR- patients (log-rank *p* < 0.001). Univariate late-DDFS cox analysis for the comparison of AR+ vs. AR- patients showed HR = 5.67 (95% CI 1.90-16.94, *p* = 0.002). No other factor showed a significant association with late-DDFS including: age (HR = 1.02, 95% CI 0.98-1.06, *p* = 0.377), histologic Grade (Grade 3 vs. 1-2 HR = 1.96, 95% CI 0.25-15.56, *p* = 0.524), stage (stage II-III vs. I, HR = 0.96, 95% CI 0.32-2.87, *p* = 0.943) and TILs (HR = 0.98, 95% CI 0.95-1.01, *p* = 0.178). However, number of events was low.

A list of cases with DDFS event and matched clinicopathological features is provided as Table 3. Moreover, exploratory additional survival analyses according to a cut-off of >10% of AR expression are reported in Supplementary Figure 1.

**Table 3:** List of cases with a DDFS event and matched clinicopathological features entered in univariate and multivariable cox regression models.

**Progressive number**	**Type of DDFS event**	**AR expression, %**	**Age, years**	**Grade**	**Stage**	**TILs, %**
1	Death	0	58	G3	Stage III	20
2	Distant relapse	0	54	G3	Stage III	5
3	Distant relapse	0	36	G3	Stage I-II	1
4	Distant relapse	0	72	.	Stage I-II	30
5	Distant relapse	0	44	G3	Stage III	10
6	Distant relapse	0	47	G3	Stage III	5
7	Distant relapse	0	57	G3	Stage III	0
8	Distant relapse	0	44	.	Stage III	15
9	Distant relapse	0	48	G3	Stage I-II	10
10	Distant relapse	0	58	G3	Stage I-II	10
11	Distant relapse	0	41	G3	Stage III	60
12	Distant relapse	0	73	G3	Stage I-II	10
13	Distant relapse	0	57	G3	Stage I-II	32
14	Distant relapse	0	50	G3	Stage III	35
15	Death	0	77	G1-2	Stage I-II	2
16	Distant relapse	0	68	G3	Stage I-II	0
17	Distant relapse	0	55	G3	Stage I-II	22
18	Distant relapse	0	30	G1-2	Stage III	5
19	Distant relapse	0	77	G3	Stage I-II	1
20	Distant relapse	0	52	G3	Stage I-II	30
21	Distant relapse	0	39	G3	Stage I-II	7
22	Distant relapse	0	50	G3	Stage I-II	5
23	Death	0	46	G3	Stage I-II	15
24	Distant relapse	0	51	G3	Stage I-II	1
25	Distant relapse	0	42	G3	Stage III	1
26	Distant relapse	0	70	G3	Stage I-II	20
27	Distant relapse	0	46	G3	Stage I-II	5
28	Distant relapse	0	46	G1-2	Stage I-II	3
29	Distant relapse	0	80	G3	Stage I-II	3
30	Death	0	73	G3	Stage I-II	25
31	Distant relapse	0	70	G3	Stage I-II	35
32	Distant relapse	0	53	G3	Stage III	30
33	Distant relapse	0	41	G3	Stage III	5
34	Distant relapse	0	61	G3	Stage III	5
35	Distant relapse	0	70	G3	Stage I-II	10
36	Distant relapse	0	52	G3	Stage III	20
37	Distant relapse	1	47	.	Stage I-II	2
38	Distant relapse	1	45	G1-2	Stage I-II	10
39	Death	1	50	G3	Stage I-II	12
40	Distant relapse	1	37	G3	Stage I-II	7
41	Distant relapse	1	45	G3	Stage III	35
42	Distant relapse	2	64	G3	Stage III	30
43	Distant relapse	5	38	G3	Stage I-II	35
44	Distant relapse	5	30	G3	Stage I-II	7
45	Distant relapse	5	52	G3	Stage I-II	0
46	Distant relapse	5	52	G3	Stage I-II	2
47	Distant relapse	20	48	G3	Stage I-II	5
48	Distant relapse	30	64	G3	Stage I-II	2
49	Distant relapse	30	74	G3	Stage I-II	10
50	Death	40	82	G3	Stage III	5
51	Distant relapse	50	42	G3	Stage III	30
52	Distant relapse	70	66	G3	Stage III	1
53	Distant relapse	75	74	G3	Stage III	5
54	Distant relapse	80	54	G3	Stage I-II	1
55	Distant relapse	85	65	G3	Stage III	1
56	Distant relapse	90	79	G3	Stage I-II	5
57	Distant relapse	90	84	G1-2	Stage I-II	7
58	Distant relapse	90	73	G3	Stage I-II	5
59	Distant relapse	95	41	G1-2	Stage III	5
60	Distant relapse	99	64	G3	Stage III	1
61	Distant relapse	99	55	G3	Stage III	10
62	Distant relapse	100	47	G3	Stage I-II	10

*Events also considered in the late-DDFS analysis are highlighted in gray. DDFS, distant disease-free survival*.

### Additional Analyses in Patients Treated With Neoadjuvant Chemotherapy

Of the 108 patients who received neoadjuvant chemotherapy, information on pathological response was available for 107 cases. A pathological complete response (pCR), defined as the absence of invasive cancer cells in the breast and axillary lymphnodes on the surgical specimen, was observed in 28% of cases (*n* = 30). The rate of pCR was similar in AR+ and AR- patients: 25.9 and 28.8%, respectively (*p* = 0.778). Tumor tissue sample from the surgical specimen obtained after neoadjuvant chemotherapy was available for AR evaluation for *n* = 60 patients without pCR (patients' flow diagram provided in Supplementary Figure 2). AR expression showed a non-significant decrease after neoadjuvant chemotherapy: mean 13% on the diagnostic core-biopsy and 10% on the paired surgical specimen (Wilcoxon signed-rank test *p* = 0.172). All those cases that were AR- on the diagnostic core-biopsy were also AR- on the surgical specimen (*n* = 43), whereas 41% of the 17 initially AR+ cases lost AR expression after neoadjuvant chemotherapy (χ^2^ *p* < 0.001).

## Discussion

In this study we showed that AR expression is associated with worse DDFS in TNBC patients treated with surgery and systemic chemotherapy. Although AR did not retain an independent prognostic value for DDFS in multivariate analysis in the total follow-up period, we found that AR expression was the only factor that resulted in a significant increase in the risk of late-DDFS event. Of note, the vast majority of events were distant relapses or deaths in patients with unresectable locoregional recurrences. Therefore, the potential confounding effect of deaths of unknown cause or not related to breast cancer (which may be relevant in studies with long-term follow up) is very limited.

We found that 30% of TNBC cases were classified as AR+, which is in line with a number of other studies (5–8). The correlation of AR+ status with other clinicopathologic characteristics such as older age, non-ductal histology, lower histologic grade, lower ki67 and lower TILs, is also consistent with other studies assessing AR by immunohistochemistry or evaluating the LAR molecular subtype (4, 9, 12, 16).

The available evidence on the prognostic role of AR for patients with early TNBC is conflicting. A recent metanalysis reported that AR expression significantly predicts for a better survival in TNBC (HR for DFS = 0.64, 95%CI 0.51-0.81 and HR for OS = 0.64, 95%CI 0.49-0.88) (13). Multivariate analysis was not available. It has to be noted that this was a study-level and not a patient-level metanalysis, including studies that were heterogeneous for methods of AR scoring, clinical cohorts characteristics, treatment and length of follow up. At least two other retrospective studies were issued after the publication of this metanalysis, reporting no association of AR with prognosis in TNBC (sample size of *n* = 130 and *n* = 182, respectively) (17, 18). In addition, two other larger studies have recently demonstrated an unfavorable prognosis for AR+ TNBC patients (8, 9). In both these studies the Dako AR441 antibody was used and the definition of AR+ in immunohistochemistry was based on the >1% cut-off, consistently with the methods applied in our analysis. Data from the TNBC subset of the prospective Nurses' Health Studies cohorts (*n* = 581) have reported, over a median follow up of 16.5 years, a significantly unfavorable breast cancer-specific survival in multivariable models for AR+ vs. AR- patients (8). In this study the prognostic impact of AR was evident in years 0–7 after diagnosis with an HR of 1.59 (95%CI 1.07–2.37) that maintained a similar value even >7 years after diagnosis, although not reaching statistical significance in this period (HR = 1.41, 95%CI 0.84–2.36). When looking at survival curves in this study, they result very similar to the ones reported in our analysis, with a separation of the curves for AR+ and AR- patients that starts around 3 years after diagnosis, supporting our findings of AR+ tumors being associated with an increased risk of late relapses. In another retrospective series of more than 300 TNBC (9), the significant association between AR+ and worse outcome was further refined by the combined evaluation of AR and forkhead-box A1 (FOXA1), a protein required for AR transcriptional activity (19). Indeed, patients with AR+/FOXA1+ TNBC showed a worse overall survival as compared to other patients in multivariable model (HR = 1.57, 95%CI 1.01-2.45) (9). Again, survival curves started to separate at around 3 years after diagnosis.

Although AR expression by itself can only be considered as a suboptimal surrogate of the molecular LAR TNBC subtype (20), our results, together with the ones by Kensler et al. and Guiu et al. are consistent with findings suggesting the association of LAR subtype with poor prognosis in TNBC (16). Potential biological reasons for this association may include: the proposed oncogenic role of AR in TNBC (11, 12) and a distinct genomic landscape including an enrichment in somatic *PIK3CA* and *AKT1* mutations (9, 16). Moreover, AR+/LAR TNBC are associated with lower TILs (4), as also shown in our study. In particular, in our work, this correlation might explain the lack of independent prognostic role of AR for DDFS in the total follow-up period when both TILs and stage are added to the multivariate model.

Anti-androgen therapies are under investigation for breast cancer in different settings (12) and phase II studies in metastatic TNBC AR+ patients have already obtained encouraging results (21–23). If further validated by other studies, our results showing that TNBC AR+ patients are at increased risk of late DDFS event may be useful in planning the future development of antiandrogen adjuvant therapies in TNBC.

With regards to the subset of patients treated with neoadjuvant chemotherapy, we did not observe different rates of pCR according to AR, however sample size was limited. The majority of data indicate that TNBC with a positive AR expression or owing to the LAR subtype achieve lower rates of pCR as compared to other TNBC patients (4, 24–26), although other studies showed conflicting results (6). The achievement of pCR is associated with long-term outcome in TNBC. Whether and to which extent the less likelihood of pCR for AR+/LAR TNBC contributes to the long-term outcome of these patients is not clear at this time (25, 26). Moreover, interpretation of results from different studies is limited by the lack of concordance between the evaluation of AR expression by immunohistochemistry and the LAR classification by gene expression. The evaluation of combined chemotherapy and antiandrogen therapy is ongoing in the neoadjuvant setting (NCT02689427).

Our study has strengths, including: the large sample size, the homogeneous treatment received by patients which is consistent with contemporary standards (all patients treated with chemotherapy and surgery, the vast majority received both an anthracycline and a taxane), the methods for AR assessment in line with the most recent studies and the length of follow up (median 81 months), allowing to uncover the impact of AR on late outcome.

Limitations of our study include the retrospective nature and the low number of events in late-DDFS analysis that imposes caution in results interpretation and further validation in additional studies.

In conclusion, our results show that the evaluation of AR in TNBC is able to identify a subgroup of patients at worse prognosis, especially for the occurrence of late events. Further validation in other studies is warranted. These data support the rationale for the ongoing evaluation of antiandrogen therapies in TNBC.

## Author Contributions

MD planned and coordinated the manuscript. VT performed the pathology assessment. VG supervised the whole writing of the manuscript. Each author participated for appropriate portions of the content. All the authors conceived the review and approved of the final analysis and results.

### Conflict of Interest Statement

MD has received fees from EliLilly for consultancy role and participation on advisory boards; fees from Genomic Health for consultancy role; fees from Celgene for participation on advisory boards. VG has received honoraria from EliLilly and Roche for participation on advisory boards, and honoraria from AstraZeneca and Novartis. The remaining authors declare that the research was conducted in the absence of any commercial or financial relationships that could be construed as a potential conflict of interest.
