# Supplementary figures and images for: Androgen Receptor Expression and Association With Distant Disease-Free Survival in Triple Negative Breast Cancer: Analysis of 263 Patients Treated With Standard Therapy for Stage I-III Disease
Source: Front Oncol. 2019 Jun 6;9:452. doi: 10.3389/fonc.2019.00452 (PMC6563384; doi:10.3389/fonc.2019.00452)

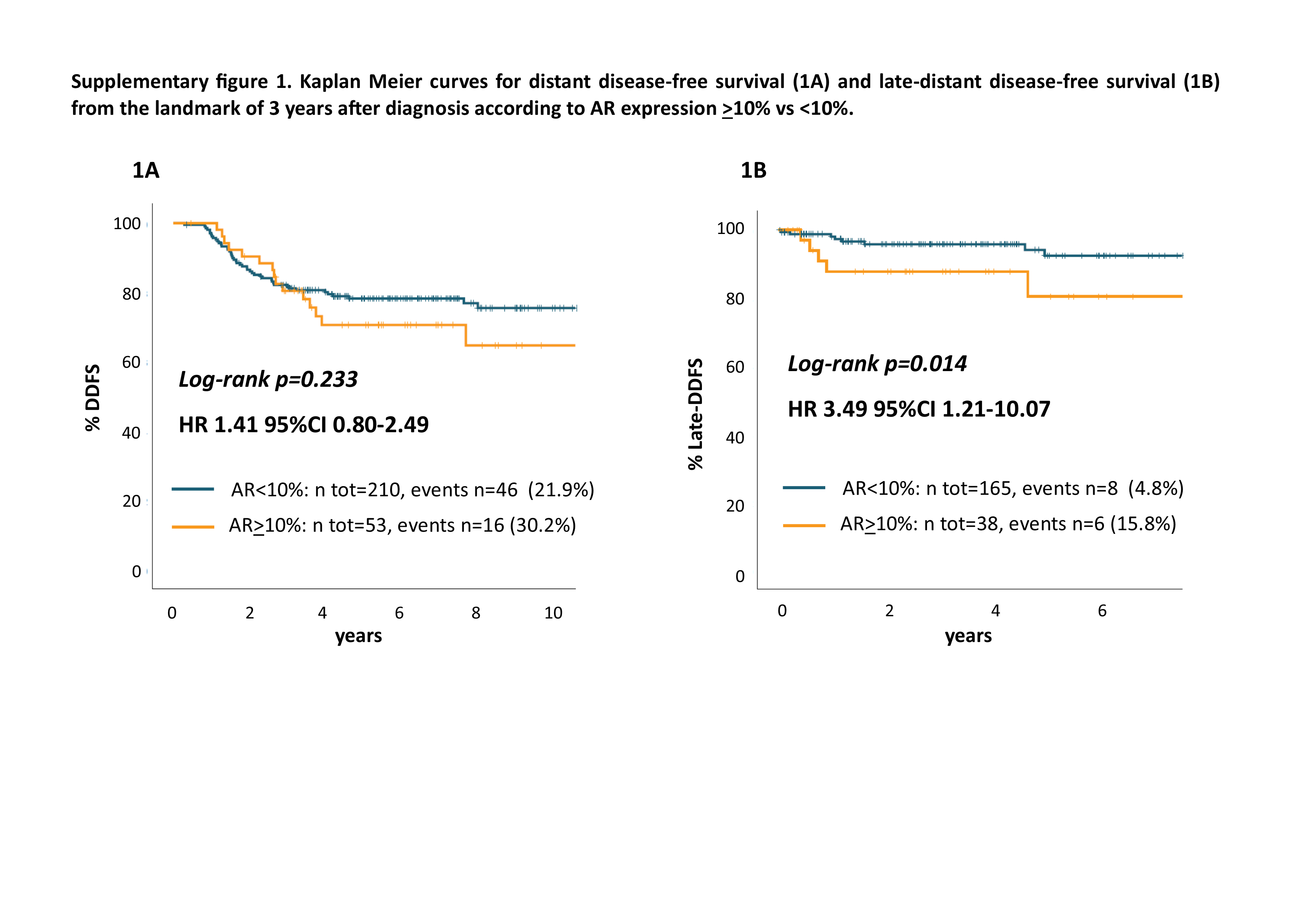

Supplement: Supplementary file 1 [file Image_1.TIF]

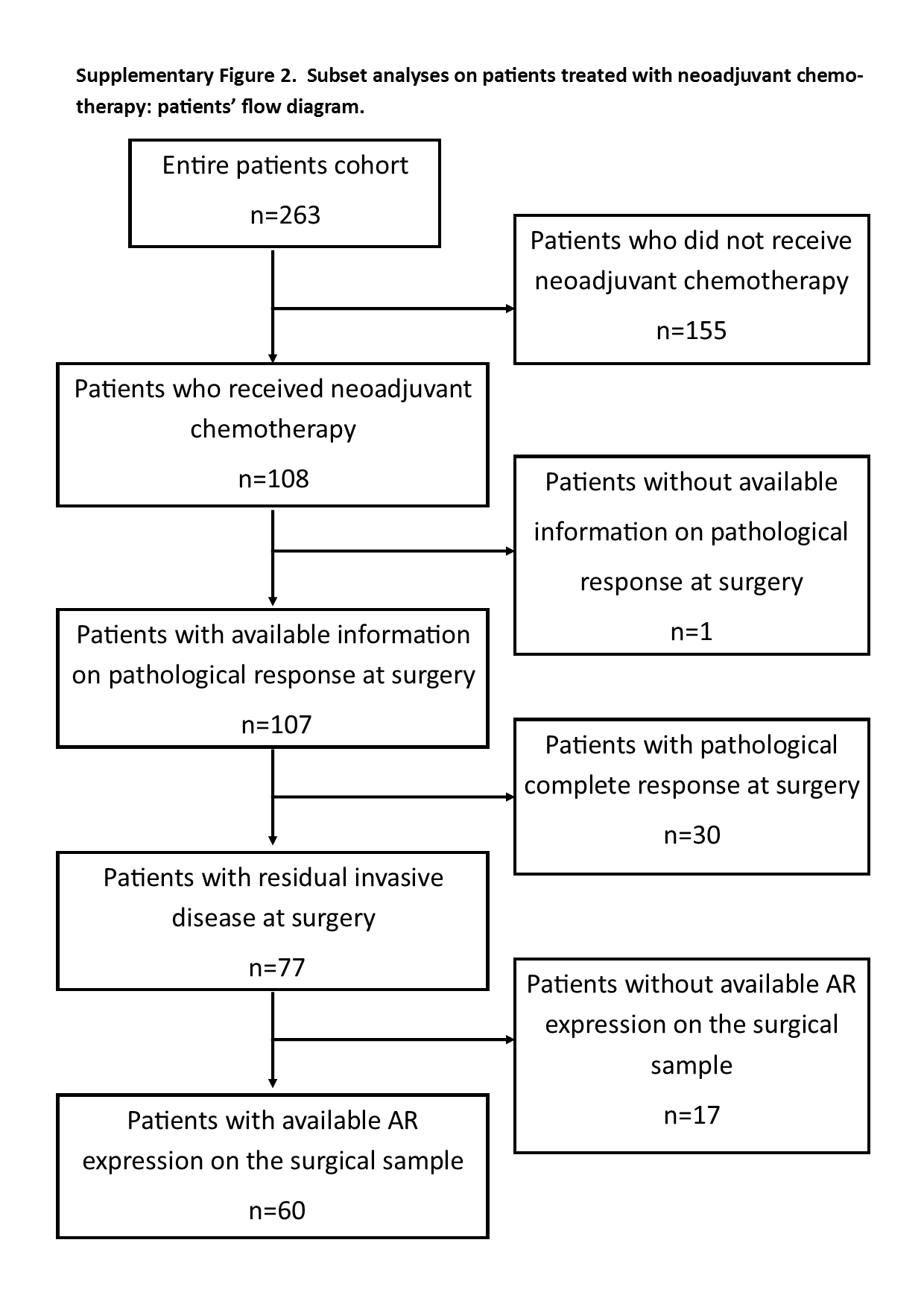

Supplement: Supplementary file 2 [file Image_2.TIF]
